# Integrating Evidence From Systematic Reviews, Qualitative Research, and Expert Knowledge Using Co-Design Techniques to Develop a Web-Based Intervention for People in the Retirement Transition

**DOI:** 10.2196/jmir.5790

**Published:** 2016-08-03

**Authors:** Nicola O'Brien, Ben Heaven, Gemma Teal, Elizabeth H Evans, Claire Cleland, Suzanne Moffatt, Falko F Sniehotta, Martin White, John C Mathers, Paula Moynihan

**Affiliations:** ^1^ Institute of Health and Society Newcastle University Newcastle upon Tyne United Kingdom; ^2^ Institute of Design Innovation The Glasgow School of Art Glasgow United Kingdom; ^3^ School of Planning, Architecture and Civil Engineering Queens University, Belfast United Kingdom; ^4^ UKCRC Centre for Diet & Activity Research (CEDAR) MRC Epidemiology Unit University of Cambridge Cambridge United Kingdom; ^5^ Institute of Cellular Medicine Human Nutrition Research Centre Newcastle University Newcastle upon Tyne United Kingdom; ^6^ Institute of Health and Society Centre for Oral Health Research Newcastle University Newcastle upon Tyne United Kingdom

**Keywords:** intervention studies, health behavior, retirement, Internet

## Abstract

**Background:**

Integrating stakeholder involvement in complex health intervention design maximizes acceptability and potential effectiveness. However, there is little methodological guidance about how to integrate evidence systematically from various sources in this process. Scientific evidence derived from different approaches can be difficult to integrate and the problem is compounded when attempting to include diverse, subjective input from stakeholders.

**Objective:**

The intent of the study was to describe and appraise a systematic, sequential approach to integrate scientific evidence, expert knowledge and experience, and stakeholder involvement in the co-design and development of a complex health intervention. The development of a Web-based lifestyle intervention for people in retirement is used as an example.

**Methods:**

Evidence from three systematic reviews, qualitative research findings, and expert knowledge was compiled to produce evidence statements (stage 1). Face validity of these statements was assessed by key stakeholders in a co-design workshop resulting in a set of intervention principles (stage 2). These principles were assessed for face validity in a second workshop, resulting in core intervention concepts and hand-drawn prototypes (stage 3). The outputs from stages 1-3 were translated into a design brief and specification (stage 4), which guided the building of a functioning prototype, Web-based intervention (stage 5). This prototype was de-risked resulting in an optimized functioning prototype (stage 6), which was subject to iterative testing and optimization (stage 7), prior to formal pilot evaluation.

**Results:**

The evidence statements (stage 1) highlighted the effectiveness of physical activity, dietary and social role interventions in retirement; the idiosyncratic nature of retirement and well-being; the value of using specific behavior change techniques including those derived from the Health Action Process Approach; and the need for signposting to local resources. The intervention principles (stage 2) included the need to facilitate self-reflection on available resources, personalization, and promotion of links between key lifestyle behaviors. The core concepts and hand-drawn prototypes (stage 3) had embedded in them the importance of time use and work exit planning, personalized goal setting, and acceptance of a Web-based intervention. The design brief detailed the features and modules required (stage 4), guiding the development of wireframes, module content and functionality, virtual mentors, and intervention branding (stage 5). Following an iterative process of intervention testing and optimization (stage 6), the final Web-based intervention prototype of LEAP (Living, Eating, Activity, and Planning in retirement) was produced (stage 7). The approach was resource intensive and required a multidisciplinary team. The design expert made an invaluable contribution throughout the process.

**Conclusions:**

Our sequential approach fills an important methodological gap in the literature, describing the stages and techniques useful in developing an evidence-based complex health intervention. The systematic and rigorous integration of scientific evidence, expert knowledge and experience, and stakeholder input has resulted in an intervention likely to be acceptable and feasible.

## Introduction

Leading international bodies in health and social care research and governance advocate the integration of stakeholder involvement in the design and development of novel health interventions [1-3]. Stakeholder input in intervention development is important to ensure that the intervention is relevant and useful for those people or groups who have or could have an interest in it. This, in turn, has the potential to maximize the acceptability and potential effectiveness of the intervention. Contemporary methods for designing products or services have moved away from using material and supplier-centered processes to more social and user-centered processes [4]; consequently, design-oriented approaches to health care innovation are being more widely recognized [5-7]. Involving relevant stakeholders as co-designers of health interventions allows the stakeholders to help define the health care problem and identify preferred intervention solutions [8-12]. Divergent and convergent thinking may result in the generation of new intervention ideas and selection of the best idea available. Intervention ideas are prototyped and explored hands-on, through sequential processes to rehearse the future [4,7]. Despite the growing use of co-design techniques in health care innovation, there is no explicit, replicable, and accepted description of their application in the development of complex health interventions.

Stakeholder involvement alone is not sufficient for effective intervention development. A range of research methods also needs to be applied, including careful consideration of existing evidence of need, and for effectiveness and cost-effectiveness of interventions to tackle the specific problem. Qualitative research provides depth of understanding to the relevant issues. Evidence-based medicine has been formally recognized as one of modern medicine’s most important milestones [13] and is applied increasingly across the fields of public health, behavioral medicine and health, and social care. Systematic and rigorous methods, including systematic reviews, meta-analyses, and meta-syntheses, for identifying and evaluating the evidence base and identifying and developing theory are key elements in the process of developing complex health interventions [14]. Quantitative and/or qualitative data are synthesized to draw conclusions about likely intervention effects and potential effect modifiers. For example, the active ingredients or features of interventions, such as behavior change techniques (BCT) associated with more positive behavior change and theory underpinning effective interventions, can be identified [15-19]. These conclusions can then be used to inform the development of novel interventions including the features that are most likely to be effective [20,21]. This systematic and theoretical approach to intervention development, along with accuracy of reporting the intervention protocol, facilitate the evaluation and replication of the intervention. While this approach to evidence synthesis is desirable, in practice it can be challenging because of the absence of established methods to guide the application of evidence to the specific population or clinical context in building a complex intervention [22,23].

Qualitative research methods help inform the development of complex health interventions [24,25]. Interviews, focus groups, and observational methods can explore the needs, attitudes, behavior, and contextual factors of the specific population and health topic under investigation [26]. The outcomes of such qualitative research can help intervention developers identify potential further intervention effect modifiers, which may inform tailoring of the intervention, thereby increasing the likelihood that the intervention will be accepted and effective.

In practice, intervention developers are likely to use deductive and inductive research methods to generate the evidence base on which the novel intervention is based. Mixed-method studies attempt to bridge the epistemological differences between quantitative and qualitative data acquisition approaches [27]. Such an inclusive approach to evidence synthesis, integrating different forms of scientific evidence, can be challenging, especially when mixed-method findings conflict [28]. Moreover, methodological guidance on how to integrate this evidence with stakeholder needs and preferences is lacking. This paper aims to fill this important methodological gap, describing and appraising a systematic and sequential approach to intervention development, drawing on techniques of co-design. Specifically, we detail the stages and techniques used to integrate quantitative (systematic review) and qualitative (interviews and focus groups) evidence, and expert knowledge and experience to engage stakeholders in a co-design process. The process is illustrated through the development of a Web-based lifestyle intervention (Living, Eating, Activity, and Planning in retirement: LEAP) to promote health and well-being of people in retirement.

## Methods and Results

### Overview

**Figure 1:**
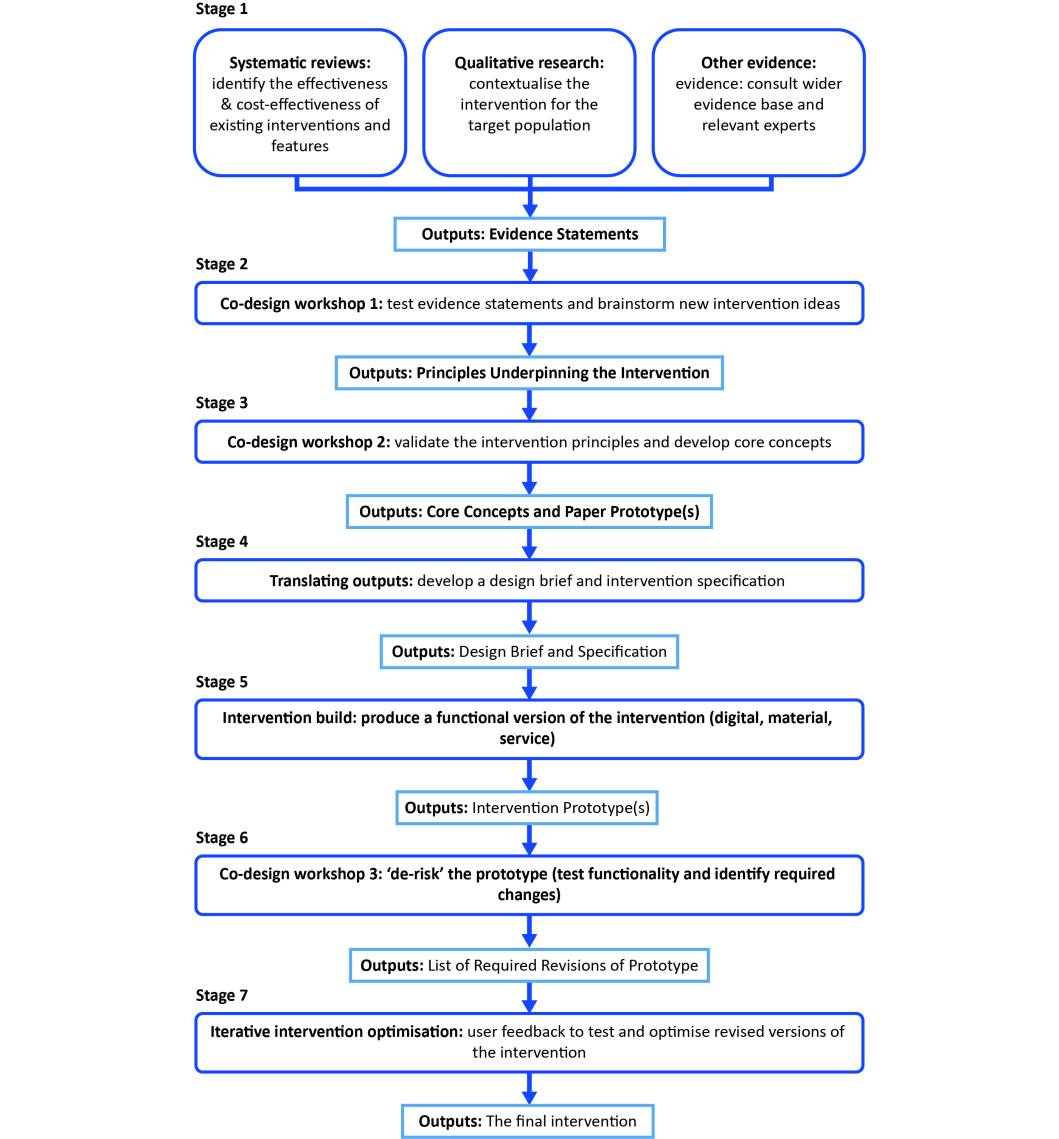
Overview of the systematic, sequential approach to intervention co-design and development.

An iterative co-design process involving sequential validation of the evidence, generation of intervention ideas, and prototyping, testing, analyzing, and optimizing the intervention was followed. This process is described as a series of stages in which each stage in the process resulted in output(s) to inform the design of the intervention. After each stage, the research team discussed and analyzed the output(s) and critically reflected on the process. Outputs from each stage were used subsequently as inputs to the next stage of development. The methods and results of each stage are therefore presented sequentially. Figure 1 displays an overview of the methods employed and outputs derived at each stage.

The context and underlying rationale behind developing a Web-based lifestyle intervention to promote health and well-being of people in retirement is presented in the following summary.

### Summary of the Intervention Development Context

Retirement from full-time work is a life transition that has been shown to be associated with changes in key lifestyle factors. Some cross-sectional and longitudinal studies have shown that people engage in more healthy behaviors with retirement [29-31]. However, the evidence is inconsistent and other studies have shown reduced physical activity (PA) [32,33], less healthy dietary behavior [34], and a loss of perceived status and purpose [35]. The population health and well-being benefits of physical activity, a healthy diet, in particular one based on a Mediterranean dietary pattern, and social engagement are well documented [36-38]. Despite recent evidence that today’s older adults are healthier than they were 10-20 years ago [39], with a globally ageing population and the accompanying increase in the prevalence of chronic ill health and morbidity [40], maintaining a healthy lifestyle into later years is vital for individual well-being and to lessen the burden on society.

As engagement with key health and social behaviors may change in retirement, the retirement transition offers a unique window of opportunity to intervene to improve health and well-being of older adults [41]. A small number of studies have delivered lifestyle interventions in the retirement transition [42-45], and systematic reviews synthesizing data from this life stage provide support for their effectiveness [46-49].

A predefined priority for the research team was to develop a personalized, scalable, sustainable, and potentially cost-effective intervention. Web-based interventions can be tailored to the individual user and may be more compatible with modes of accessing information and support (eg, by mobile phone) in future cohorts of older people. They also have greater potential for wide scale use in the target population. As such, the possibility of a Web-based intervention was a planned consideration of the research team.

**Figure 2:**
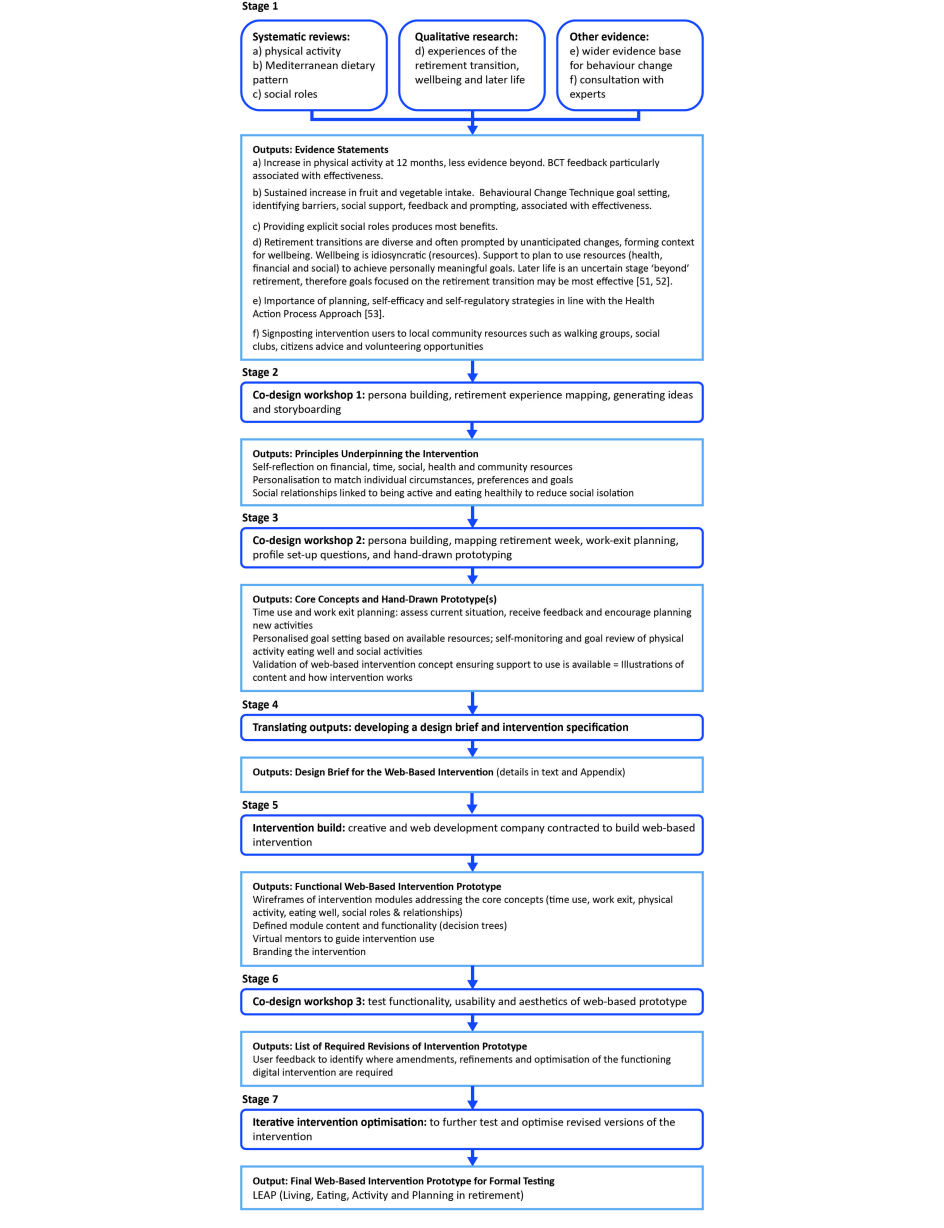
Applied example: integrating systematic review, qualitative research and other evidence with stakeholder engagement in a co-design process to develop the LEAP intervention.

Figure 2 details the process by which the sequential intervention development approach (detailed in Figure 1) was applied to develop LEAP, a Web-based lifestyle intervention in retirement. The specific outputs of each stage in the development of LEAP are presented.

### Stage 1: Compiling the Evidence Base

#### Stage 1 Procedure

Evidence from systematic reviews, qualitative research, and other sources, including the wider evidence base for behavior change and consultation with relevant experts, was summarized by the research team.

#### Stage 1 Analysis

The evidence was recorded as a list of “evidence statements” [50] that informed the aims and content of co-design workshop 1 (stage 2).

#### Stage 1 Outputs

The evidence statements are listed in Figure 2 and descriptions of the evidence are enlarged upon in Multimedia Appendix 1. The systematic reviews, which in part underpinned these statements, have previously been published [18,19,47-49].

### Stage 2: Co-design Workshop 1

#### Stage 2 Participants

A total of 42 stakeholders participated in co-design workshop 1. Participants included 12 members of the research team (6 workshop facilitators and 6 scribes), 22 adults aged 55 years or over (9 males) as potential intervention users, and 8 health and social care professionals (3 males) from the voluntary sector and public health organizations, whose role was related to improving health and well-being of people in retirement. The research team included health researchers from a range of disciplines involved in improving health and well-being in older people and with combined expertise in design, behavior change, public health, physical activity, nutrition and dietetics, and social gerontology.

Older adults from local older people’s forums were sampled purposively to represent men and women at different stages in the retirement transition and from diverse socioeconomic backgrounds.

#### Stage 2 Procedure

The aim of stage 2 (co-design workshop 1) was to determine the face validity of the evidence statements and brainstorm new intervention ideas, which were informed and inspired by the evidence statements. In preparation, discussion among the research team identified that the qualitative work provided the context in which the intervention would be built. The qualitative work emphasized individual experiences through retirement; retirement was commonly experienced as a process rather than a discrete event. The systematic review evidence and the theoretical framework of the Health Action Process Approach provided recommendations regarding intervention content, modalities, and timings. Through these discussions, the research team identified the need for co-design techniques that could combine descriptive, context-rich narratives with more discrete evidence regarding health and social behaviors.

The workshop took place in a university space. Participants were divided into small groups, each comprising older adults, health and social care professionals, one facilitator, and one scribe. Facilitators guided the structure and timing of the workshop, and scribes recorded participants’ comments and ideas.

The first technique used in the workshop was the co-design of a persona. As retirement is both a process and idiosyncratic, “persona building” [51] was a useful technique in order to orient each group to real world issues in retirement. Each group of workshop participants was assigned a different persona with a description that was a composite of different accounts and experiences of participants in the qualitative study and from clinical experience of the research team. The personas represented male and female older adults from a range of socioeconomic positions (see Multimedia Appendices 2 and 3). Each workshop group considered one of the behaviors examined in the systematic reviews [47-49]. For example, Jeff was said to be physically inactive but had enjoyed being active when he was younger.

The second technique was “experience mapping” [52] of the retirement transition. To generate intervention ideas including considering when it would be most needed, accepted, and potentially effective, we mapped different retirement pathways identified in the qualitative study. Each group considered the possible key stages in the persona’s retirement transition such as “Jeff gets made redundant,” “Jeff retires,” and “Jeff gets a part-time job.” This technique allowed participants to discuss how the persona might feel during the retirement transition, reflecting on their own experiences. Each group generated intervention ideas that would help tackle the specific health, social, or resource challenges of the persona in each scenario. In particular, groups were asked to consider Web-based intervention ideas. Drawing on evidence statements regarding the importance of the local environment and community resources to facilitate behavior change, groups were presented with a map of the persona’s local area and asked to think about whether local resources could support the intervention ideas generated.

“Wild cards” representing random events that might disrupt the retirement story were introduced to mimic the unpredictable nature of real life and challenge the participants to consider whether these events would alter the retirement story. The wild cards also provided opportunities to discuss how specific intervention features, such as behavioral change techniques, could be incorporated in the persona’s retirement pathway.

The third technique was “storyboarding” [53]. This allowed the group to pull their different ideas together to form a new intervention to support an ideal retirement experience for their persona. The intervention ideas from each group provided an outline of a potential intervention, which was defined to include its name, how it would be signposted or advertised, features to encourage initial and longer-term engagement, and the lifestyle behaviors it would help to promote.

#### Stage 2 Analysis

Shortly after the workshop, the facilitators prepared detailed notes to capture group discussions and describe how the participants tackled each activity, reflecting on the key insights and ideas discussed within each group. Analyzing the outputs of the workshop activities revealed recurring design ideas (ie, broad goals for the intervention), which became the “principles underpinning the intervention.” The intervention principles were derived from validating the acceptability and importance of the evidence statements with potential users, and therefore the principles reflected the context in which the intervention would be built and ideas for intervention content, modality, and timing. These principles were further explored and developed by stakeholders at subsequent workshops (co-design workshops 2 and 3, described below) to generate more tangible ideas for products and features for the intervention. The method used to analyze the facilitator notes and workshop materials resembles thematic analysis [54], a technique that allows for the identification of repeated patterns of meaning. As used here, it captured design ideas in addition to themes.

#### Stage 2 Outputs

The outputs of co-design workshop 1 were the potential intervention ideas from each of the six groups. The potential intervention components, resources for the intervention, and the key design priorities were identified by the research team. The most common themes and features in the intervention ideas formed the following intervention principles:

1. *Self-reflection* on financial, time, social, health, and community resources; the intervention should provide practical assistance in planning or structuring activities focusing on key life events rather than age;

2. *Personalization* to individual circumstances, preferences, and goals, providing a flexible intervention with individual feedback and tailored support from a mentor;

3. *Social relationships* linked to being physically active, eating healthily, in order to reduce risks of social isolation, promote a sense of social support, and share experiences in an engaging way.

### Stage 3: Co-design Workshop 2

#### Stage 3 Participants

A total of 20 stakeholders participated in co-design workshop 2: 6 members of the research team (3 facilitators and 3 creative facilitators) and 14 older adults (6 males). Older adults were recruited from local forums as before.

#### Stage 3 Procedure

Co-design workshop 2 aimed to obtain user feedback on the intervention principles derived from co-design workshop 1. Feedback was used to assess face validity of the principles and to develop the core intervention concepts. The workshop took place in a local community meeting space and lasted 4 hours including refreshment breaks. Participants were divided into three groups, each of which was led by a workshop facilitator. Each group was also supported by a creative facilitator with design expertise, who sketched the intervention ideas as they were being generated and facilitated the development of hand-drawn prototypes of potential interventions using paper Web browser templates. Web browser templates were used to explicitly explore the acceptability of a Web-based intervention.

In preparation for this workshop, the design expert identified further co-design techniques to facilitate the presentation and interaction with specific aspects of the intervention principles using Web browser visual materials and prompts. “Prototyping” was a key technique used to communicate ideas, which enabled the progression of thinking through physical making, a safe space for failure leading to faster learning, and encouragement and permission to explore new behaviors [55].

Validation of the self-reflection intervention principle was conducted using mock-ups of a work transition tool with interactive graphs and texts. This tool, which supported individuals to reflect on possible work exits (eg, retiring fully, reducing hours) or re-entry (eg, returning to employment) as identified in the qualitative work [56], was developed in each workshop group.

To further explore the personalization intervention principle, participants were asked to consider what questions the intervention platform should ask to learn about the persona’s attitudes and habits in relation to the target lifestyle behaviors. The answers to these questions would shape how the intervention could be personalized to meet the persona’s needs, circumstances, and goals. Participants wrote the questions on cards, placed them in a natural conversational order, and considered options for how they could be presented (eg, written text, video clips, and images).

The co-design technique of persona building was used to further explore and validate the social relationships intervention principle, while also providing further opportunity to explore the act of planning included in the self-reflection principle. Groups mapped a typical week during retirement, focusing on the absolute and relative time the persona engaged in lifestyle behaviors related to being physically active, eating healthily, and spending time with other people.

#### Stage 3 Analysis

Detailed notes capturing each group’s discussion, how the participants tackled each activity, and key feedback on the intervention principles were produced by the facilitators. Using thematic-based analysis as in stage 2, facilitator notes and the hand-drawn prototypes of potential interventions were analyzed to identify recurring design ideas and intervention user requirements, and to define the core intervention concepts, which reflected the intervention principles and the target lifestyle behaviors of physical activity, healthier eating, and social roles.

#### Stage 3 Outputs

The outputs of co-design workshop 2 were core intervention concepts and the hand-drawn *prototypes* of novel interventions, which served to document how the intervention principles were validated through user feedback. The “work transition” and “mapping a retirement week” tools were evaluated as enabling self-reflection; providing feedback on financial, time, and social resources (including considering how social relationships can be linked with other activities during a week); and facilitating future goal setting and planning. Seeing when new activities could take place was deemed to be extremely valuable, providing insight into potential spare time. It also served to prompt people to set boundaries (eg, ensuring they did not overcommit to looking after grandchildren) and goals.

A core set of personalization questions were defined whose answers would allow the intervention to be tailored to individual needs and goals. The result was a low-fidelity (limited function) prototype of the user registration component of the intervention, with each webpage hand-drawn on a deck of paper templates.

The majority of participants welcomed a Web-based intervention, acknowledging the benefits of having access at home and at convenient times, and the intrinsic ability of a Web-based intervention to be tailored to the individual. However, there were concerns that some individuals may feel unsupported by technology and consequently disengage from the intervention, further reinforcing the need for support from a mentor outlined in the personalization design principle. Potential cost-effectiveness and scalability of the intervention were predefined priorities of the research team. Therefore, providing access to someone to support use of the intervention, such as a health care assistant, was considered unfeasible. Consequently, the role of a virtual mentor within the Web-based intervention, who could help the user explore retirement transition options and lifestyle behaviors, was explored and positively appraised.

The research team identified common themes and features of the hand-drawn prototypes that related to the target lifestyle behaviors to form the following core intervention concepts: (1) time use and work exit planning as an opportunity to assess current financial, time, and social resources, receive feedback, and encourage the planning of new activities, (2) personalized goal setting based on identified available resources, self-monitoring of behavior, and regular reviewing of lifestyle goals, and (3) a Web-based intervention as an acceptable mode of delivery, providing that support to use is available.

### Stage 4: Translating Outputs Into a Design Brief and Specification

#### Stage 4 Procedure

The aim of this stage was for the research team to examine, critically evaluate, and translate the outputs from the previous stages into a detailed design brief and specification document to inform the intervention build.

#### Stage 4 Analysis

The evidence statements (stage 1), design principles (stage 2), hand-drawn prototypes, and core intervention concepts (stage 3) were examined for recurring design ideas and intervention requirements across all outputs. These ideas and requirements were evaluated critically by the research team for concurrence with the team’s predefined priorities, the intervention development context, and their suitability to support the promotion of the target lifestyle behaviors.

#### Stage 4 Outputs

The output was a design brief and specification document detailing the aim of the intervention and the design features it should include (see Multimedia Appendix 4). The design brief stated the need for an interactive website including a set of intervention tools to support people to have a healthier and more fulfilling retirement. The design specification detailed the following design features that the intervention should include: personalized, scalable, sustainable, interactive, digital, user flow through the intervention, and visually and functionally engaging. The following intervention sections or modules to be included were also detailed: user profile, work-exit and cost of living, time and activity planner, physical activity, eating well, and social relationships.

### Stage 5: Intervention Build

#### Stage 5 Procedure

The aim of this stage was to produce a functional version of the intervention prototype. This involved a tendering process to identify a Web development company that would support the building of a functional Web-based intervention prototype. The design brief and specification were included in the tender. The research team worked closely with the contracted company throughout the process, holding regular face-to-face meetings to discuss emerging ideas for presenting the intervention content, to order and structure the intervention modules, and to maximize user engagement with the intervention.

#### Stage 5 Outputs

Wireframes for the intervention modules, detailed module content, and decision trees guiding user flow through the intervention were developed. Wireframes are simple images that show how a website and its webpages are structured and how the content is arranged. A set of six “virtual mentors” connected, if desired, to audio files recorded by local actors to provide cultural links were also developed to guide and support users through the intervention. The final output of this stage was a functioning Web-based intervention prototype for testing and optimization with stakeholders (see Multimedia Appendices 5-14).

### Stage 6: Co-design Workshop 3

#### Stage 6 Participants

A total of 37 stakeholders participated in co-design workshop 3: 8 members of the research team as facilitators and 29 older adults (12 males). Older adults were recruited from local forums.

#### Stage 6 Procedure

The aim of the third and final co-design workshop was to “de-risk” the prototype [57] through testing intervention functionality and identifying necessary modifications using a cognitive walkthrough activity [58]. The final workshop took place in a university space. Participants were divided into small groups, each of which was led by a workshop facilitator. The intervention de-risking techniques focused on exploiting user experience testing. Participants were provided with a tablet and asked to use the intervention with the aim of testing its functionality, usability, and aesthetics. Feedback, queries, technical and functional issues that participants expressed were recorded by the group facilitator on printed screenshots of each page of the intervention. The technique of persona building was used as the vehicle for the group to navigate the intervention from the perspective of the persona.

#### Stage 6 Analysis

The feedback and issues identified by each group were collated by the facilitators. Technical and functional issues were added to a list of required revisions to the intervention prototype. Other feedback, such as comments relating to the design, esthetics, or content of the intervention, was considered by the research team to ascertain whether the revisions were feasible and essential.

#### Stage 6 Outputs

The output of this stage was a comprehensive list of revisions to the intervention prototype required to improve user experience and acceptability of the intervention. Identified revisions to the prototype included (1) refining color contrasts and font size, (2) revising text content, order, and position, (3) including an intervention overview page, a dashboard summarizing the parts of the intervention with which the user has already engaged, and a diary summarizing the user’s activities scheduled for the following weeks, (4) adding progress bars for questionnaires, and (5) providing options to hear the mentor’s voice and viewing the time planner as a calendar or pie chart. An optimized functioning intervention prototype was produced following the amendments and refinements.

### Stage 7: Iterative Intervention Optimization

#### Stage 7 Participants

A group of 30 representatives of stakeholders (potential intervention users, researchers, and health and social care professionals) provided feedback on the revised intervention prototype.

#### Stage 7 Procedure

The aim of this final stage was for the revised intervention prototype to be further tested by stakeholders to identify additional ways to improve and refine the intervention. This stage adopted an iterative testing, user feedback, and intervention refinement process whereby optimization occurred in parallel with testing to ensure that new or revised features were also tested. The research team liaised closely with the Web development company to ensure that optimization occurred promptly and efficiently.

#### Stage 7 Outputs

The output was a final prototype Web-based intervention, ready for formal field testing in a pilot randomized controlled trial (to be reported elsewhere). The intervention was named LEAP (Living, Eating, Activity, and Planning in retirement). Table 1 [16,18,19,38,56,59-64] presents a summary of LEAP intervention modules, tools and interactive features, and the evidence on which each element was based.

### Ethical Approval

This work was conducted as part of the LiveWell program. Ethical approval was acquired from Newcastle University Ethics Committee (No 00423). Informed consent was obtained from participants in the qualitative study. The workshops were based on a co-design methodology where all stakeholders (research team, older adults, and health professionals) held shared “power” in the development of the new intervention. No personal data were collected, thus ethical consent to participate in the workshops was not obtained. However, informed consent was obtained for the purpose of photographically recording the activities at each workshop.

**Table 1:** The LEAP features and modules, and the objective(s), tools, and evidence on which they were based at different stages of the intervention development process.

LEAP section/ module	Objective(s)	LEAP tools	Evidence base
User profile	Register the user and set preferences		Qualitative research found that retirement transitions and available resources are idiosyncratic. The user profile supports the tailoring of LEAP to address the variable nature of retirement transitions [56,59]. Preliminary information about the user’s retirement stage, physical activity, diet, and social circumstances is captured and used to tailor the introduction of the related module.
Intervention overview (Multimedia Appendices 5-7)	Provide an overview of the modules and their objectives, and guidance on the general functions and features of LEAP. Set personal preferences (mentor, email bulletin).	Interactive carousel overview of modules.	Co-design workshop 3 identified the need for an overview to provide a guide to the intervention modules and tools, including emphasizing the intended dip-in and dip-out nature and user-determined flow through the intervention.
		Opt-in for LEAP to be personalized and supported by a virtual mentor. One of eight mentors could be chosen with the option of hearing their voice or reading the text.	Co-design workshop 1 identified the need for a mentor to support user journey through the intervention. This idea was appraised positively during co-design workshop 2. Virtual mentors were developed and optimized during co-design workshop 3.
		Opt-in to receive weekly email bulletin summarizing recent usage of LEAP and prompt revision of goals and plans (BCT: follow-up prompts, goal review).	Systematic review of dietary interventions found that the BCT follow-up prompts was associated with greater intervention effectiveness [19]. Self-regulation prompts for action control [60] is an effective behavior change strategy [61].
Time module (Multimedia Appendix 8)	Reflect on current and desired future time use over the retirement transition.	Interactive calendar or pie chart time planner.	Qualitative research indicated that assistance to reflect on current and future time use was important. Considering how time might be spent in retirement (eg, additional care of relatives, unstructured time) might help identification of personalized goals (eg, a need for a structured role or activity) and potential barriers/facilitators to goal achievement [56]. This module provides space and tools for the user to think through the possibilities and opportunities for lifestyle behaviors, goals, and aspirations.
			Co-design workshop 1 found that a time reflection tool would be useful. Co-design workshop 2 found that providing a choice of how the time planner tool is presented (calendar or pie chart style) is desirable and that this module was valuable to “set the scene” for other modules, where activities could be considered and scheduled.
Changing Work module (Multimedia Appendix 9)	Consider financial and work situation as participant moves through the retirement transition.	Interactive bar charts and graphs to visualize different retirement trajectories and the effect on income and expenditure.	Qualitative research indicated that finances and modes of work transition (eg, full to part-time, fully retire, retired to part-time) are idiosyncratic and lay the foundation for different retirement experiences and the adoption and maintenance of lifestyle choices [59]. This module allows the user to consider their circumstances and opportunities to engage in new activities (eg, continuing working reduces available free time but the continued income could mean can retire earlier).
			Co-design workshop 1 showed that a work exit tool was appraised positively by potential users. Co-design workshops 2 and 3 further developed and refined this tool.
Moving More module (Multimedia Appendix 10)	Awareness of current physical activity level. Opportunity and tools to engage in self-regulation of PA.	Pedometer to facilitate self-monitoring, goal setting including step-count, receive feedback, schedule activities, identify barriers, and revisit and review step and activity goals (BCT self-monitoring, goal setting behavior and outcome, goal review, action planning, barrier identification).	Physical activity was a predefined target behavior.
			Systematic review of physical activity suggested that the BCT providing feedback was associated with greater long-term effectiveness [18]. Evidence for the effectiveness of other self-regulation BCTs to promote PA, in line with the Health Action Process Approach [16,62-64].
			Co-design workshop 1 confirmed that the BCTs of self-monitoring, goal setting, and action planning were acceptable to stakeholders and potentially valuable for most. Co-design workshops 2 and 3 further developed and refined this module.

Being Social module (Multimedia Appendix 11)	Explore potential benefits of having a meaningful occupation/ role or spending time with significant others.	Interactive social relationship mapping, social role case studies and schedule activities (BCT: action planning).	Social connectedness was a predefined target behavior.
			Systematic review of social roles suggested that interventions providing explicit roles with group support were effective [48]. The social roles tool provides resources to explore explicit roles.
			Participating in social relationships has been identified in the literature as key to well-being in later life [38].
			Qualitative research confirmed the importance of social relationships but did not identify a clear opportunity for intervention [56].
			Co-design workshop 2 identified a potential intervention mechanism through a relationship reflection tool, supporting by structured suggestions for maintaining and building social relationships.
Eating Well module (Multimedia Appendix 12)	Awareness of current diet and provision of information to make diet more Mediterranean in style.	Mediterranean diet quiz and feedback, goal setting, recipe book, schedule trying a new recipe, identify barriers, and revisit and review goals (BCTs: information about consequences of behavior, goal setting behavior and outcome, goal review, action planning, barrier identification).	A predefined target behavior.
			Systematic review of Mediterranean dietary patterns suggested that the BCTs of goal setting, identifying barriers, feedback, and follow-up prompts were associated with greater effectiveness [19].
			Co-design workshop 1 identified the need for a self-assessment tool to appraise fit between current diet and Mediterranean eating pattern, with personalized feedback and suggestions to improve.
			Co-design workshop 2 confirmed acceptability of the module’s core functions, including personal goal setting, feedback, and follow-up prompts, in line with BCTs identified in systematic review [19]. A meal planner and recipe guide were also judged acceptable.
			Co-design workshop 3 confirmed acceptability of the barrier identification and coping planning features. Stakeholders suggested improvements to interface usability and clarity.
Diary (Multimedia Appendix 13)	Schedule PA, trying a new Mediterranean diet recipe or social activity for the current and following week.	Intelligent design remembers previously named significant others and prompts to add them to the scheduled activity (BCT: action planning).	This feature arose in co-design workshop 3 and was developed subsequently as a way to summarize the activities a user had scheduled and link with the weekly email bulletin to encourage revisiting the intervention to update data, get feedback, revise goals and plans, and schedule new activities. Evidence for the effectiveness of self-regulation behavioral change techniques to promote health behaviors, in line with the Health Action Process Approach [16,18,19,62-64].

## Discussion

### Principal Findings

This paper’s key contribution is providing a description of a systematic, sequential approach to integrating scientific evidence from systematic reviews, qualitative research, and expert knowledge and experience with stakeholder involvement to develop an evidence-based complex health intervention. We have detailed the stages employed including the co-design techniques used and the outputs produced, and have demonstrated the application of the approach in the development of LEAP, a Web-based lifestyle intervention for people in the retirement transition. Here we provide a critical appraisal of this approach.

### Strengths and Limitations

The approach presented in this paper follows and complements the Medical Research Council (MRC) guidance for the development of complex health interventions [14]. As advocated in this guidance, our approach applied systematic and rigorous methods to identify and evaluate the evidence base and the theoretical basis for a novel intervention. In addition, we have described the practical stages and methods required to integrate this evidence with stakeholder input. Specifically, we have utilized co-design methodology to facilitate stakeholder engagement and input, which can be modified and refined to suit the specific intervention context and target population. Our approach adds to recent studies using co-design techniques for health care innovation [5-7] providing a concrete example of how to apply these methods in the development of a Web-based lifestyle intervention in retirement.

Our intervention development approach follows seven distinct stages, each of which has the following strengths, limitations, and challenges. Stage 1, “compiling the evidence base,” is an essential component of intervention development [14], but depending on the size and extent to which the evidence base has been interrogated previously, this stage can be resource intensive, which may be a barrier for projects with scarce resources. In the example of developing LEAP, there was limited existing evidence for the effectiveness of lifestyle interventions in retirement and on experiences of the retirement transition. The wider evidence base for behavior change and knowledge of local resources with which the intervention could link were evidence sources that already existed yet required explicit interrogation in relation to the intervention objectives.

A challenge faced during stage 2 co-design workshop 1 involved the use of personas. Some stakeholders struggled with this concept initially but when the purpose of using a persona was further explained by researchers, stakeholders engaged with the process. In addition, many stakeholders found that the wild cards, used to assess how specific BCTs could be incorporated in the persona’s retirement pathway, were too abstract and difficult to grasp, thus limiting the assessment of their potential value and acceptability in the intervention. However, allowing stakeholders to explore how BCTs work in practice using a prototype intervention at a later stage in the intervention development process (stage 4) was found to be more effective.

The possibility of a Web-based intervention was a planned consideration of the research team as the aim of the LiveWell program, set out in the funding agreement, was to develop a personalized, scalable, sustainable, and potentially cost-effective intervention. However, a Web-based intervention was suggested as a potential mode of delivery for the intervention by several of the groups of stakeholders in this workshop. A strength of using co-design techniques is that they can be used to support stakeholders to explore the evidence base for a novel intervention but they can also be used to stimulate the creation of other intervention ideas. Although a Web-based intervention was a planned consideration of the research team, we were cognizant of the potential limitations related to the so-called digital divide and subsequent health inequalities in intervention access and use [65]. This issue was further explored in the formal pilot evaluation of LEAP (reported elsewhere).

The success of stage 2 co-design workshop 1, stage 3 co-design workshop 2, and stage 4 translating outputs into a design brief and specification, relied critically on contributions from our design expert. During stage 2, the design expert identified established co-design techniques, including persona-building and storyboarding, to facilitate the development of persona narratives within the scope of the qualitative evidence, and as a means to punctuate these narratives with opportunities to pursue the specific lifestyle behaviors identified in the systematic reviews. They also ensured that the evidence statements, written in scientific terms, were translated into plain English and presented in a visually engaging way so that they were accessible and interesting for all participants. During stage 3, the design expert guided the creative facilitators to sketch the intervention ideas generated and prototype potential interventions. When testing prototypes, the researchers observed that the level of fidelity of the prototypes was important; sketched ideas were easier for participants to engage with than more detailed mock-ups of one part of the intervention were, which was interpreted as a finished product inviting little useful feedback [66]. These visual aids served both as prompts for discussion in the workshop and as illustrations of design ideas for use in stage 4.

The challenge of stage 4 was to ensure that recurring design ideas contained in the prototypes were translated into specific features for tools, modules, or requirements of the intervention. Here, the design expert supported the production of a design brief and specification that reflected the outputs from previous stages while also detailing the intervention content and function that should be included in the intervention build.

A challenge of stage 6 co-design workshop 3 was that some user feedback suggested revisions to the prototype that were deemed unfeasible or non-essential by the research team and therefore were not addressed in the revisions. For example, idiosyncratic feedback about the value of particular modules or features indicated that not all parts of the intervention would be useful to all users. Rather than trying to anticipate which parts of the intervention would be most valuable to a user on the basis of their user profile, the team decided to emphasize in the intervention overview that LEAP is designed to allow a user to choose which modules or features to engage with, in an order of their choice. This would also allow an individual to revisit other parts of the intervention at a later date when perhaps their needs and priorities had changed.

The challenge of translating requirements from multiple perspectives and evidence sources (i.e., people with experience of retirement, organizations, and subject experts) alongside the scope and stated aims of the research program required that pragmatic compromises were made. Decisions were taken through discussion by the research team. In addition, the contracted Web-development company had many design decisions to make in the functional intervention build that were not directly influenced by co-design stakeholders until the prototype testing stages.

A strength of our approach is that it draws on the diverse skills of a multidisciplinary team with expertise in a range of research methodologies, including systematic reviewing, qualitative enquiry, and intervention co-design and development. Teams also need the subject expertise required to develop the particular intervention, which in our case included PA, nutrition and dietetics, social gerontology, and information technology expertise.

### Comparison of our Intervention Development Approach With Other Approaches

Our sequential approach fills an important methodological gap in the complex health intervention development literature providing the description and appraisal of how to integrate systematic review, qualitative research, and other evidence with stakeholder engagement in a co-design process. Other approaches have advocated the integration of user perspectives in intervention development, demonstrating the importance of conducting qualitative research with a wide range of people from the target user populations at every stage of intervention development, from planning to feasibility testing and implementation [25,67]. In addition, the application of co-design techniques in health care intervention development has been demonstrated [8-11]. Our approach values the role of qualitative research and stakeholder input in intervention development but also details *how* to integrate systematic review evidence in the process, which is an important component of the MRC guidance [14]. Moreover, we provide detailed information on the stages and methods required to follow the approach to develop an intervention that is not only evidence-based but also fits the needs of intervention stakeholders, thereby increasing the likelihood of the intervention being acceptable and feasible. A 6-step guide [68] attempts to fill the methodological gap in the literature by providing a guide of how to develop public health interventions from defining the problem and identifying the modifiable causal and contextual factors through to collecting preliminary evidence of effectiveness. Our approach complements this guide describing the specific methods and co-design techniques that can be employed at each step.

As illustrated by our example of developing LEAP, the approach we have tested enables a clearly documented description of the intervention development process including the evidence on which each intervention feature/characteristic was based and the potential causal mechanisms of change in terms of BCTs used (see Table 1). Documenting the process in this way ensures that the intervention can be clearly described and reported, facilitating future replication. Thus our approach supports researchers to conform to the Template for Intervention Description and Replication (TIDieR) intervention reporting guidelines [69] and to develop an intervention logic model or “theory of change,” which can direct an evaluation of the effectiveness of the intervention as advocated in the MRC guidance on process evaluation of complex interventions [70].

### Implications of our Approach

The approach we present provides a sequential description of the methods needed to pursue evidence and theory-based complex health intervention development. The co-design techniques we employed, namely persona building [56], experience mapping [59], storyboarding [60], and prototyping [43], originate from product and service design adopting a social and user-centered process [4]. Co-design techniques have been used to involve stakeholders as co-designers in health care innovation and intervention development [5-11]. Our approach adds to this growing body of literature providing an explicit and replicable description of how to apply the techniques using the example of the development of LEAP, a complex lifestyle intervention for people in the retirement transition.

As discussed, the approach can be labor and time intensive. In the illustrative example of developing LEAP, a large proportion of the project timeline was attributed to delivering the outputs of stage 1. Conducting high-quality systematic reviews is a lengthy and resource-intensive process, which in our example, included additional work to identify the associations between intervention features and effectiveness. This was a necessary stage in the process as current systematic review evidence of interventions for PA, Mediterranean dietary patterns, and social roles for people in retirement did not exist. Where recently conducted, high-quality systematic reviews exist, these can be used to develop the evidence statements to inform intervention co-design (stage 2), maximizing the time and resources available for the later stages of designing, building, and de-risking the intervention.

### Further Work

We have demonstrated that a sequential approach can be applied to the development of a Web-based lifestyle intervention for people in retirement. Further work is needed to apply this approach to other areas of health intervention development. Further application and refinement of this approach would help build evidence about its utility and acceptability. This in turn could support the development of formal guidance on this process.

The final output of our approach to intervention development is a functional prototype (in our case, the Web-based intervention LEAP) ready for formal testing to ascertain the effectiveness of the intervention. Web-based interventions have significant promise to reach the rapidly expanding older adult population who are increasingly becoming routine Internet users [71] and have been shown to have positive effects on lifestyle behaviors, including PA, in older adults [72-74]. The feasibility and acceptability of LEAP has been formally tested in a pilot randomized controlled trial (NCT02136381), which will be reported elsewhere. The pilot data will be used to inform the design of a definitive evaluation of the effectiveness and cost-effectiveness of LEAP.

### Conclusions

This paper fills an important methodological gap in the complex health intervention development literature by describing and appraising a systematic, sequential approach to the co-design and development of an evidence-based complex health intervention. Using the example of the development of the LEAP intervention, we have illustrated the application of this approach and detailed the stages and techniques followed, integrating quantitative and qualitative evidence (derived from systematic reviews and qualitative research), expert knowledge and experience, and stakeholder involvement.

## Appendix

Multimedia Appendix 1 Further description of outputs of Stage 1 that underpinned evidence statements.

Multimedia Appendix 2 Example of persona used in Stage 2 Meet Helen.

Multimedia Appendix 3 Example of personal used in Stage 2 Meet Jeff.

Multimedia Appendix 4 Summary of design brief and specification.

Multimedia Appendix 5 Screenshot of LEAP welcome page.

Multimedia Appendix 6 Screenshot of mentor selector.

Multimedia Appendix 7 Screenshot of mentor overview.

Multimedia Appendix 8 Screenshot of My Time Module.

Multimedia Appendix 9 Screenshot of Changing Work Module.

Multimedia Appendix 10 Screenshot of Moving More Module.

Multimedia Appendix 11 Screenshot of Being Social Module.

Multimedia Appendix 12 Screenshot of Eating Well Module.

Multimedia Appendix 13 Screenshot of Diary.

Multimedia Appendix 14 Screenshot of dashboard.

## Abbreviations

BCT: behavior change technique
LEAP: Living, Eating, Activity, and Planning in retirement
MRC: Medical Research Council
PA: physical activity
