# Supplementary material for: Integrating Evidence From Systematic Reviews, Qualitative Research, and Expert Knowledge Using Co-Design Techniques to Develop a Web-Based Intervention for People in the Retirement Transition
Source: J Med Internet Res. 2016 Aug 3;18(8):e210. doi: 10.2196/jmir.5790 (PMC4989122; doi:10.2196/jmir.5790)
Supplement: Multimedia Appendix 1 [file jmir_v18i8e210_app1.pdf]

Further description of outputs of Stage 1 that underpinned evidence statements

a) a meta-analysis of the long-term effectiveness of physical activity interventions and moderator analyses of the association between features of interventions (modes of delivery and behaviour change techniques (BCTs)) and effectiveness concluded that physical activity interventions can be effective in the long-term [49] and providing feedback may be a key component [18];

b) a meta-analysis of the effectiveness of dietary interventions and moderator analyses of the association between features of interventions (modes of delivery and behavioural change techniques) and effectiveness concluded that interventions promoting fruit and vegetable consumption and, to a lesser extent, those promoting decreased meat consumption, were effective [47] and were more likely to report the use of behavioural change techniques of goal setting, barrier identification, social support, feedback and prompting [19];

c) a narrative synthesis of the effectiveness of interventions providing explicit social roles concluded that social interventions that provide explicit and meaningful social roles for people in retirement are likely to yield the greatest benefits for health and wellbeing [48];

d) a qualitative study involving 48 workers/retirees aged 53-77 years of diverse socioeconomic status about their experiences of retirement transition, wellbeing and later life concluded that an intervention in retirement should be tailored to the unanticipated changing circumstances surrounding retirement and provide support for the identification of personalised short to medium term goals and available health, financial and social resources, and for planning how to achieve goals [51, 52];

e) the wider evidence base for behaviour change resulted in the identification of the Health Action Process Approach [53] as a theoretical framework recognising the importance of planning, self-efficacy and self-regulatory strategies (action control) for behaviour change, which was consistent with evidence statements ‘a-d’. Additional behavioural change techniques, such as coping planning, were identified from the Health Action Process Approach with the potential to promote intervention maintenance effects;

f) consultation with community providers and organisations concluded that the intervention should sign-post users to local resources such as walking groups, social clubs, citizens advice and volunteering opportunities.
