# Supplementary figures and images for: Integrating Evidence From Systematic Reviews, Qualitative Research, and Expert Knowledge Using Co-Design Techniques to Develop a Web-Based Intervention for People in the Retirement Transition
Source: J Med Internet Res. 2016 Aug 3;18(8):e210. doi: 10.2196/jmir.5790 (PMC4989122; doi:10.2196/jmir.5790)

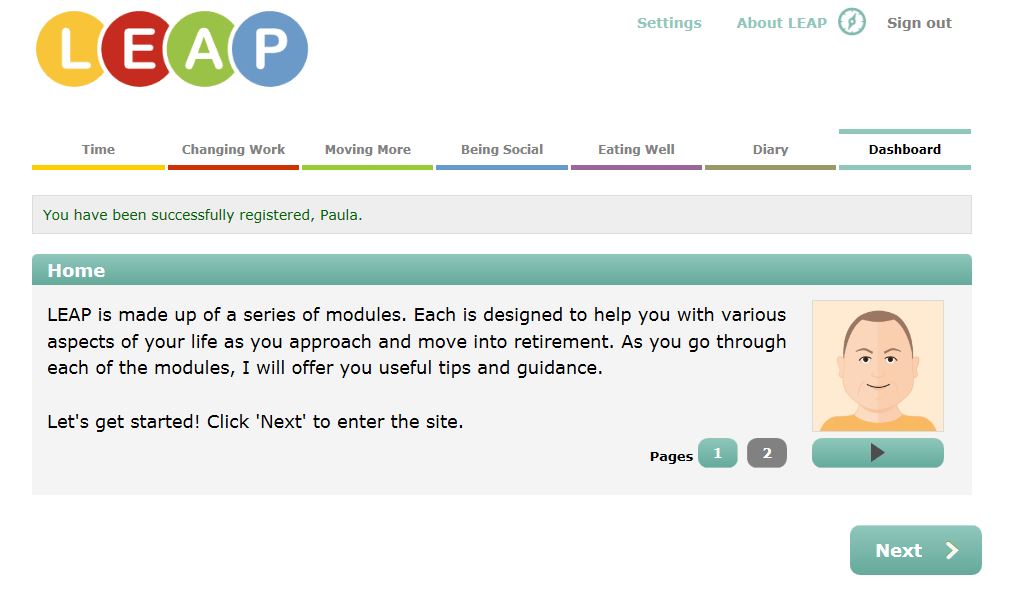

Supplement: Multimedia Appendix 2 [file jmir_v18i8e210_app2.JPG]

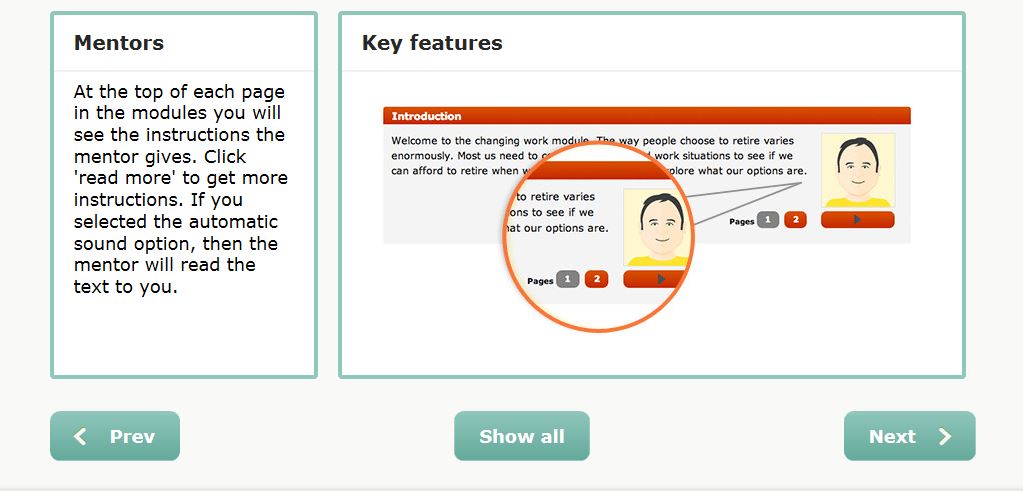

Supplement: Multimedia Appendix 3 [file jmir_v18i8e210_app3.JPG]

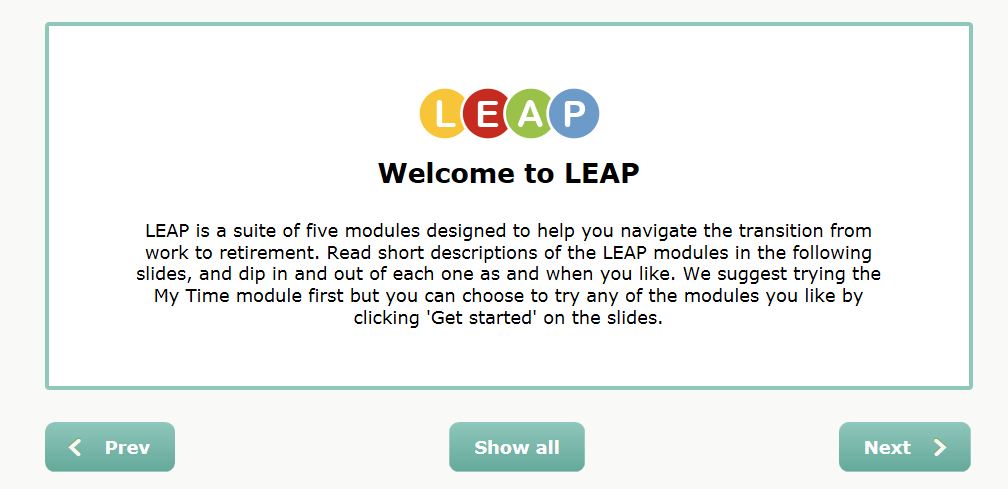

Supplement: Multimedia Appendix 5 [file jmir_v18i8e210_app5.JPG]

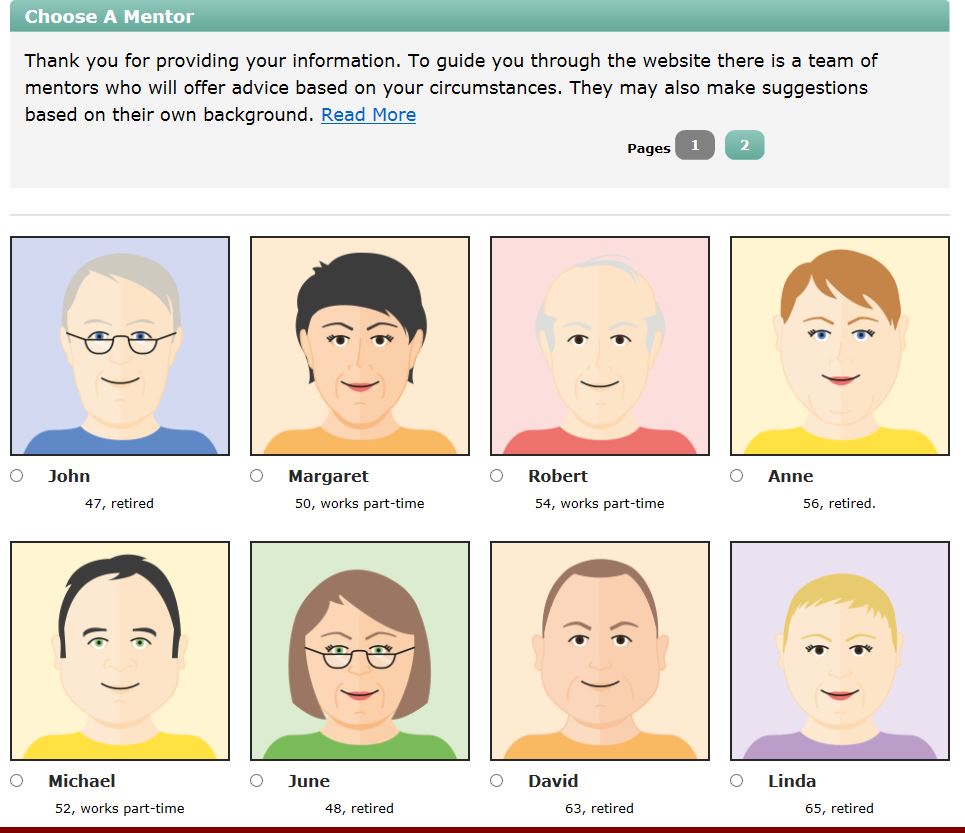

Supplement: Multimedia Appendix 6 [file jmir_v18i8e210_app6.JPG]

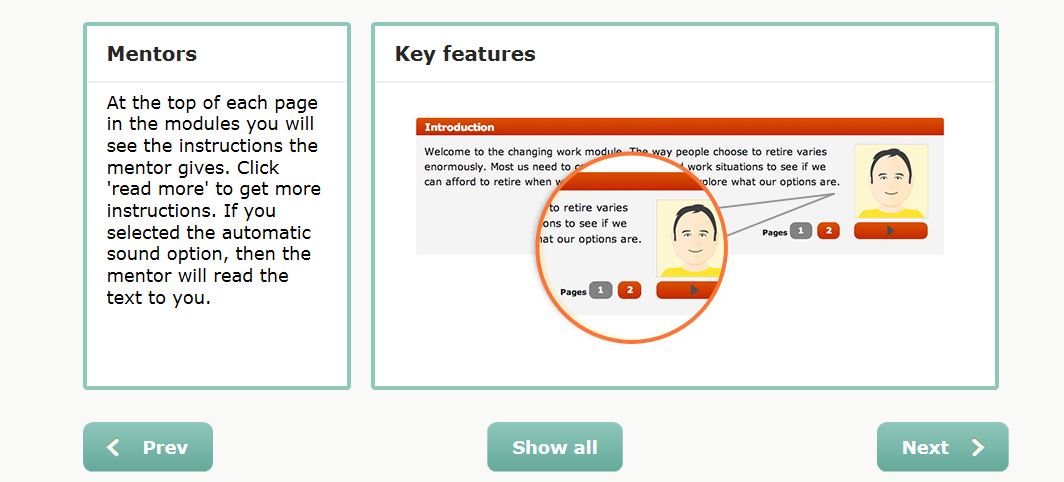

Supplement: Multimedia Appendix 7 [file jmir_v18i8e210_app7.JPG]

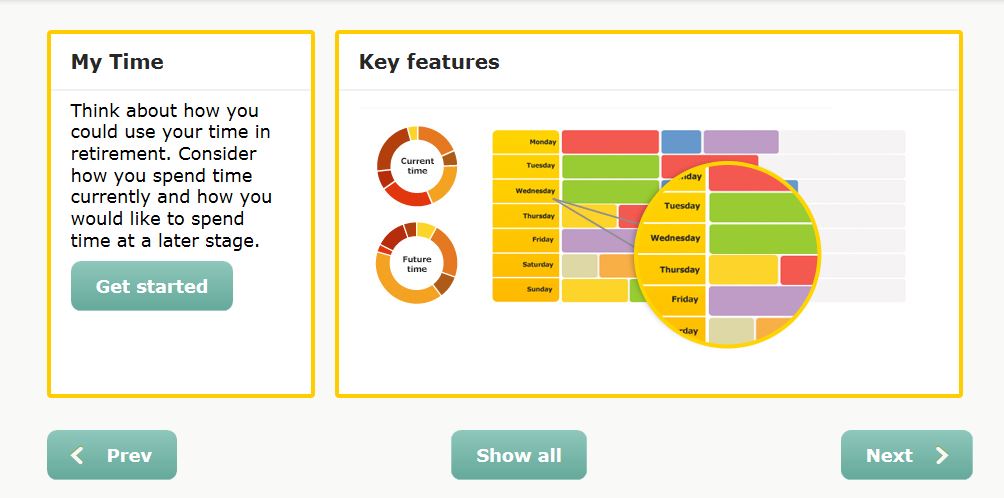

Supplement: Multimedia Appendix 8 [file jmir_v18i8e210_app8.JPG]

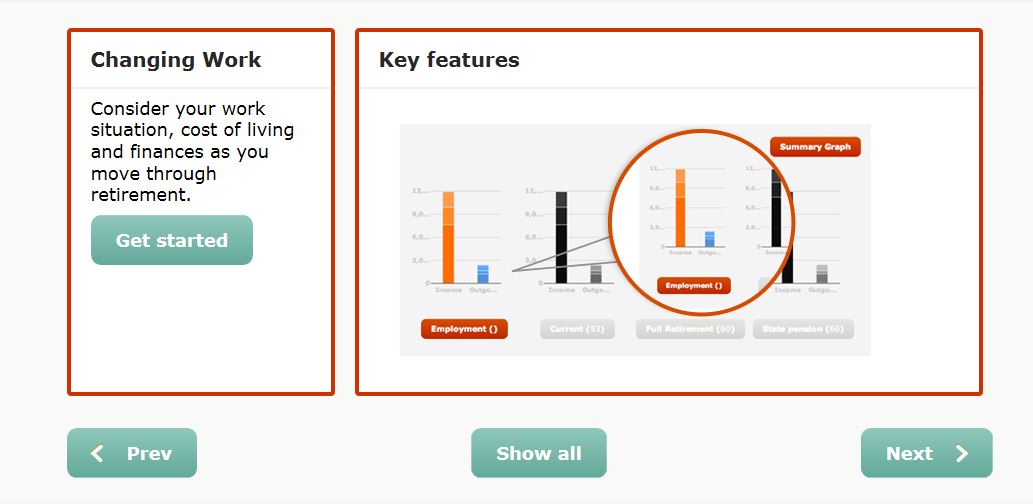

Supplement: Multimedia Appendix 9 [file jmir_v18i8e210_app9.JPG]

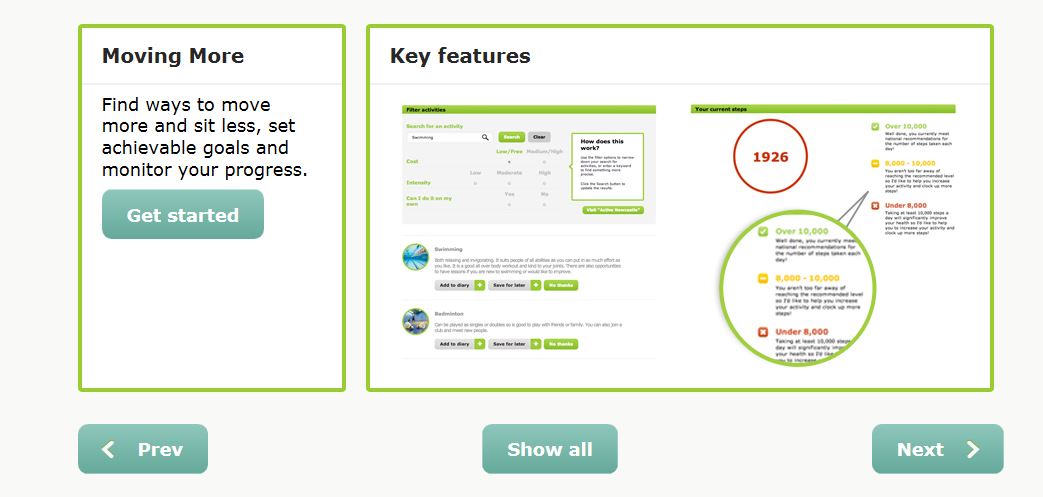

Supplement: Multimedia Appendix 10 [file jmir_v18i8e210_app10.JPG]

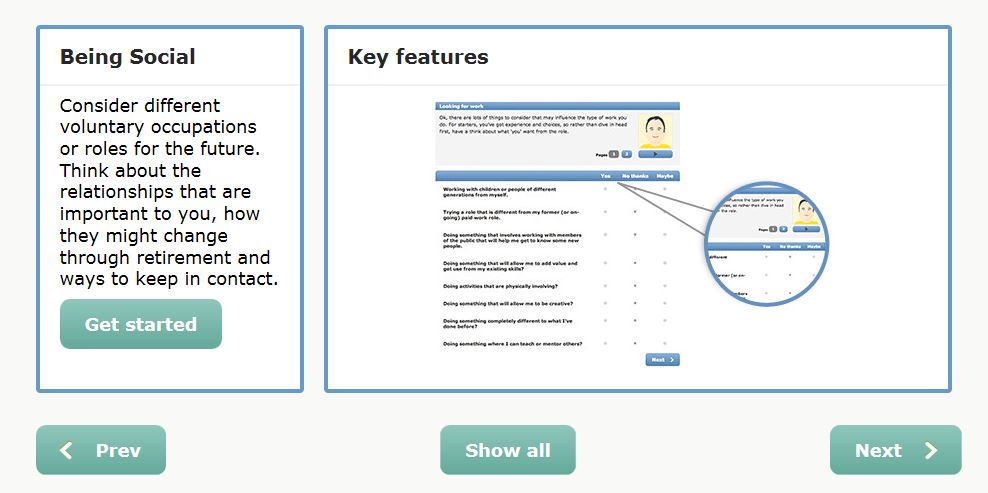

Supplement: Multimedia Appendix 11 [file jmir_v18i8e210_app11.JPG]

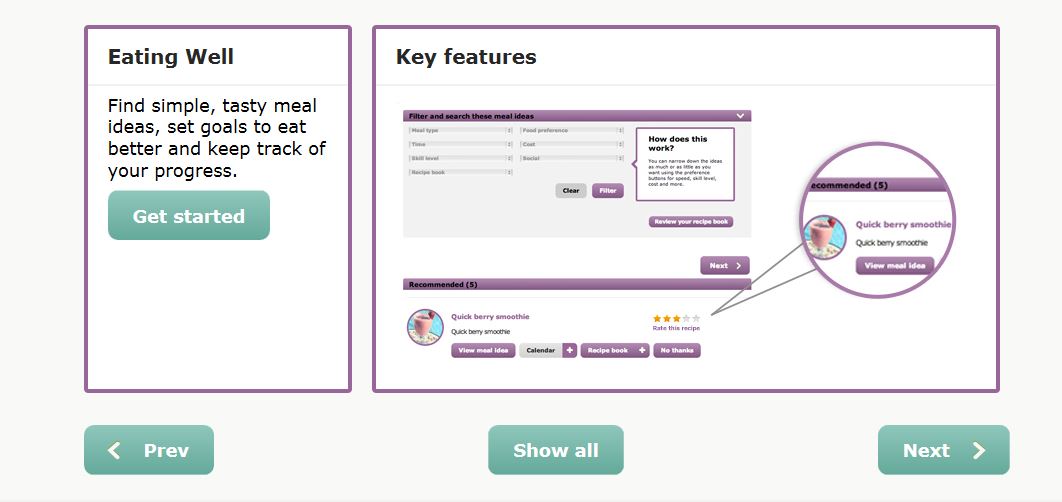

Supplement: Multimedia Appendix 12 [file jmir_v18i8e210_app12.JPG]

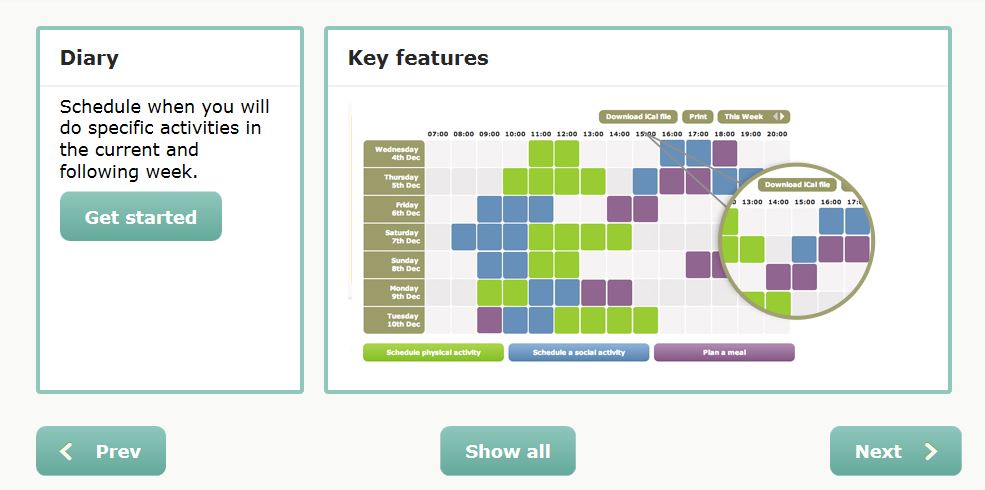

Supplement: Multimedia Appendix 13 [file jmir_v18i8e210_app13.JPG]

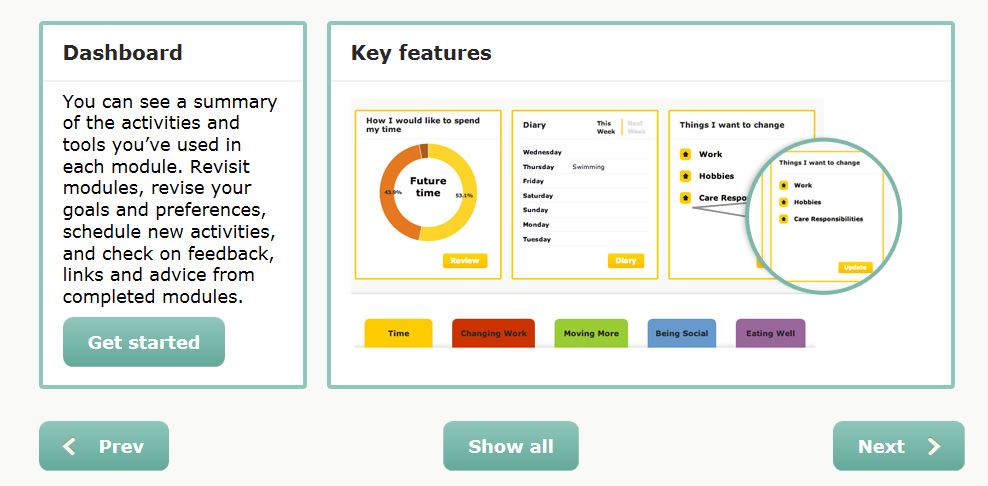

Supplement: Multimedia Appendix 14 [file jmir_v18i8e210_app14.JPG]
