# Supplementary material for: Integrating Evidence From Systematic Reviews, Qualitative Research, and Expert Knowledge Using Co-Design Techniques to Develop a Web-Based Intervention for People in the Retirement Transition
Source: J Med Internet Res. 2016 Aug 3;18(8):e210. doi: 10.2196/jmir.5790 (PMC4989122; doi:10.2196/jmir.5790)
Supplement: Multimedia Appendix 4 [file jmir_v18i8e210_app4.pdf]

Supplementary Material 2. Summary of Design Brief and Specification

The Brief: Retirement from work can be accompanied with significant lifestyle changes. Physical activity, healthier eating and social activities are all important lifestyle behaviours. We want to develop an interactive website with a set of intervention tools that support people to have a more healthy and happy retirement

Intervention Specification

The intervention must include the following *design features*:

Personalised: targeted to the needs and preferences of individuals and groups.

Scalable: suitable to be delivered in the community to large numbers of people at minimal cost

Sustainable: to help sustain desired behaviour change in the long term and the intervention itself can be sustained at low cost by whatever organisation commissions it.

Interactive: users will be actively involved in developing the resources the website provides. For example, users will be able to interact with intervention content and share it with others.

Digital: the intervention suite must be deliverable via a web-based platform, in the first instance, accessible from desktops, tablets and mobile phones.

Flow: an algorithm underpinned by defined decision trees will guide but not dictate a user's chosen pathway through the intervention modules. The system must be flexible and adaptable. Interventions modules should be inter-connected, built in a way that allows adding further modules in the future and updating modules to incorporate new evidence.

Visually and Functionally Engaging: the intervention must strike a balance between being attractive and engaging, and based on scientific evidence.

The intervention must include the following *modules and tools*:

Log in and Personal Profile: Password protected personal profile allowing archiving and retrieval (e.g., displays progress against target). This should include data acquisition and storage functions.

Work-exit and Cost-of-Living: To explore different work exits or retirement patterns and subsequent cost-of-living, and to prompt the user to seek advice on their financial situation.

Time and Activity Planner: To visualise current and desired patterns of time-use, identify challenges to health and wellbeing, and consider lifestyle goals.

Physical Activity: to establish current physical activity levels and support maintained physical activity changes (goal setting, action planning, goal review, feedback and barrier identification).
